# Effect of supplementation continuously in pullet and early lay period with *Bacillus subtilis* and yeast cell wall on the intestinal morphology, bone parameters, and egg quality of hens

**DOI:** 10.3389/fvets.2025.1584627

**Published:** 2025-04-24

**Authors:** Hang Gao, Jinzhi Xiao, Qi Zhang, Hutianlin Man, Xingyi Tang, Zhouyuan Wang, Rendong Fang, Sha Jiang

**Affiliations:** ^1^Joint International Research Laboratory of Animal Health and Animal Food Safety, College of Veterinary Medicine, Southwest University, Chongqing, China; ^2^Kunming Hemeihua Feed Limited Company, Kunming, China

**Keywords:** *Bacillus subtilis*, yeast cell wall, intestinal morphology, egg quality, young hens

## Abstract

The aim of this study was to investigate effect of *Bacillus subtilis* (BS), yeast cell wall (YCW), and their combination on intestinal and bone development and egg production of young hens. A total of 160, one-day-old Hy-Line Sonia chicks were randomly allocated into 4 treatments of 8 replicate cages of 5 birds each. Treatments were arranged in a 2 (0 and 0.5 g/kg of BS) × 2 (0 and 2 g/kg of YCW) factorial arrangement for a duration of 170-d as follows: (1) regular diet (Control group); (2) the regular diet plus 0.5 g BS/kg (BS group); (3) the regular diet plus 2 g YCW/kg (YCW group); and (4) the regular diet plus 0.5 g BS/kg and 2 g YCW/kg (BS + YCW group). One bird from each of the 8 replicate cages per treatment were randomly taken for sampling at d 110 and 170, respectively. Results indicated that there were different effects of BS and YCW on bird organ development and innate immune. YCW supplementation increased thymus index and serum concentrations of IgM of hens (*p* < 0.05). In addition, an interaction was observed between YCW diet and age on serum IL-6 concentrations (*p* < 0.05), mainly because YCW birds had the highest serum IL-6 concentration at d 110. Dietary supplementation with BS reduced the crypt depth in the duodenum and jejunum with an increased ratio of villus height to crypt depth (*p* < 0.05) in the duodenum, jejunum, and ileum. However, a YCW × age interaction on the jejunal villus height existed, mainly because non-YCW diet had the lowest jejunal villus height at d 170 (*p* < 0.05). Both BS and YCW enhanced egg weight, eggshell thickness and yolk color (*p* < 0.05), while YCW improved albumen height and Haugh unit (*p* < 0.05). There was no treatment effect on measured bone parameters except that YCW birds had lager tibial diameter (*p* < 0.05) at d 170. The results indicate that dietary BS and/or YCW improve the intestinal and bone development and immune status of young hens, which may contribute to the increased egg quality during the early sexual maturity stage.

## 1 Introduction

Antibiotics, as growth promotors, have commonly been used over the past decades to combat infections and to improve growth performance in poultry production (1). However, due to public health and food safety threats, the use of antibiotics in food-producing animals has been banned in several regions worldwide (2). Prebiotics and probiotics have been widely used as alternatives to antibiotics owing to their ability to improve gut health and enhance production performance in poultry as well as other farm animals (3). Probiotics are living bacteria (direct-fed microbials) that, when ingested in adequate amounts, confer health benefits to the host by promoting the growth of beneficial intestinal bacteria (4). Prebiotics are indigestible or unabsorbable carbohydrates, functionally promoting the metabolism and proliferation of beneficial bacteria in the gastrointestinal tract (GIT), thereby improving host health. To some extent, prebiotics often work synergistically with probiotics (5). However, the diverse range of probiotics and prebiotics, coupled with their occasional inefficacy when used independently (6), highlights the need to develop effective probiotic-prebiotic combinations specifically designed for poultry production. Such combinations would help to meet the growing demands of customers for safe and sustainable poultry products.

*Bacillus subtilis* (BS), a gram-positive bacterium belonging to the genus *Bacillus*, is widely used as a probiotic in poultry management due to the stability and heat resistance of its metabolically dormant spores (7, 8). It has been shown that dietary addition of BS improves the feed-to-egg conversion ratio and enhances yolk weight and eggshell strength in 65-week-old Leghorn layers (9). Similarly, supplementation of high doses of BS increased albumen height and Haugh unit in laying hens at 19–48 weeks of age (10). In addition, BS reduces intestinal damage in necrotizing enteritis models in broiler chickens, thereby improving growth performance (11). However, some other findings suggest that BS has no significant effect on egg production, average egg weight, and gut morphology, but merely regulates the cecal microbiota toward a healthier composition (12). The yeast cell wall (YCW) derived from brewer’s yeast through industrial processing is an odorless light-yellow powder composed primarily of mannans and *β*-glucans, which is a natural microbial polysaccharide with excellent bioactivity and nutritional value. Dietary supplementation with YCW has been shown to improve broiler gut health by upregulating the expression of intestinal tight junction-related protein genes and enhancing intestinal barrier function (13). YCW supplementation also reduces the severity of necrotizing enterocolitis, including decreased mortality and morbidity (14) by mitigating cell death, apoptosis, and innate inflammatory responses (15).

Although there are studies involving BS and YCW in poultry, the study on their combined effects on pullets during growing to lay period is scarce. The growing period is the most critical and vulnerable stage in the life cycle of a laying hen, as that during this life stage, the GIT, skeletal system, and other internal organs undergo rapid development with alterations in immune and neuroendocrine functions (16). Enhancing intestinal absorption and bone formation during the growing period is critical for ensuring a steady onset and prolonged maintenance of a peak laying cycle (17). We hypothesized that BS and/or YCW might have effects on improvement of the gut and bone status in pullets and early stage laying hens, which would result in better laying performance. Therefore, the experiment was carried out to evaluate the effects of BS, YCW, and their combination on the production performance, intestinal development, and bone parameters of pullets and early stage laying hens.

## 2 Materials and methods

### 2.1 Birds, diet, and experimental design

All procedures of this experiment were approved by the Animal Ethics Committee and performed by following the animal guidelines of the Southwest University, Chongqing, China (The permission number: IACUC-20200726-11).

A total of 160 one-day-old Hy-Line Sonia chicks (Chongqing Huayu Yaoxin Agricultural Technology Co., Ltd.) were randomly allocated to 4 groups, with 8 replicate pullet cages (70 cm × 70 cm × 30 cm) of 5 birds each (n = 8). A 2 × 2 factorial experimental design was applied for a duration of 170-d: two levels of BS (0 and 0.5 g/kg) were crossed with two levels of YCW (0 and 2 g/kg). The dietary treatments were: (1) regular diet (Control group); (2) regular diet + 0.5 g/kg of BS (BS group); (3) regular diet + 2 g/kg of YCW (YCW group); and (4) regular diet + 0.5 g/kg of BS + 2 g/kg of YCW (BS + YCW group). The BS, containing 2 × 10^11^ CFU/g, and the YCW, containing *β*-glucan (≥30%) and mannan-oligosaccharides (MOS, ≥20%), were provided by Angel Yeast Co., Ltd. (Yichang, China). The regular diet was provided by Charoen Pokphand Feed Co., Ltd. (Chongqing, China) and formulated to meet the nutrient requirements of Chinese feeding standard (NY/T33-2004). The birds were fed a starter diet (analyzed nutrient levels: crude protein, 20.1%; crude fiber,4.41%; crude ash, 6.10%; calcium, 0.95%; total phosphorus, 0.64%; Lysine, 0.91%; and Methionine + Cysteine,0.74%) from d 1 to 42; a grower diet (analyzed nutrient levels: crude protein, 16%; crude fiber, 4.20%; crude ash, 6.30%; calcium, 0.95%; total phosphorus, 0.60%; Lysine, 0.80%; and Methionine + Cysteine, 0.61%) from d 43 to 112; and a laying diet (analyzed nutrient levels: crude protein, 16.50%; crude fiber, 4.81%; crude ash, 11.10%; calcium, 3.90%; total phosphorus, 0.55%; Lysine, 0.85%; and Methionine + Cysteine,0.65%) from d 113 until d 170. At d 113, birds were transferred from pullet cage to laying cage (40 cm × 35 cm × 35 cm) with 2 birds per cage (the remaining birds were used for commercialization, n = 8) to continue the experiment until d 170. The birds from each pullet cage in the pullet period remained together as experimental unit when transferred to the laying cage. Feed and water were provided ad libitum during the entire experiment and the environmental temperature program was followed by the recommendation of the Hy-Line Sonia Commercial Layer Management Guide.

### 2.2 Sample collection

One bird from each of the 8 replicate cages per treatment was randomly taken for sampling at d 110 and 170, respectively (*n* = 8). After taken the body weight, each bird was anesthetized with pentobarbital sodium (30 mg/kg of body weight), consequently blood was collected via cardiac puncture, killed by cervical dislocation, and then the thymus, spleen, bursa, glandular and muscular stomach were collected for calculating each organ’s relative index (the organ relative index = absolute organ weight (g)/body weight (g) × 100%). The blood samples were kept at room temperature for 2 h, then centrifuged at 2,000 × g for 10 min. at 4°C. Each collected serum was divided into two aliquots and stored at −20°C for future tests. In addition, 3 cm samples around the midsegment of each of the duodenum, jejunum, and ileum were separately collected and preserved in 10% buffered formalin for subsequent analyses. Furthermore, on d 170, the right femur and tibia of each sampled bird were collected and stored at −20°C for future analyses. Concurrently, on d 166–169 (about 24 weeks of age), eggs were collected daily for egg production and egg quality analysis.

### 2.3 Serum inflammatory factors and immunoglobulin analysis

The concentrations of tumor necrosis factor alpha (TNF-*α*), interleukin (IL)-6, and IgM were measured by an automatic microplate reader using double antibody sandwich ELISA according to the manufactory manuals of each kit (Xiamen Huijia Biotechnology Co., Ltd., Fujian, China).

### 2.4 Intestinal histology analysis

Approximately 1 cm segments of the formalin-fixed intestinal samples from the duodenum, jejunum, and ileum were processed separately. The samples were dehydrated using a graded ethanol series, transparentized with xylene, embedded in paraffin, and then sectioned at 3 μm thickness using a sliding microtome (Leica RM2235, Leica microsystems, Wetzlar, Germany). The sections were stained with hematoxylin and eosin. The sections were observed and photographed under a microscope (BX53 Olympus, Japan), and villus height and crypt depth were measured using Image J software, and then villus height to crypt depth (V/C) ratios were calculated. For each sample, three non-consecutive sections with three fields per section, at least 10 villus-crypt units per sample, were analyzed and then the average values were calculated.

### 2.5 Bone performance parameters analysis

Each collected right leg was stripped of all soft tissues (skin, muscle, and fascia) for collecting the femur (n = 8) and tibia (n = 8). The length and midshaft diameter of the femur and tibia were measured using a vernier caliper (PD-301, Pro’skit, China). Each measurement was repeated three times, and the average value was recorded. The femur and tibia were placed in the bone densitometer (Dual-energy X-ray bone densitometer InAlyzer, Medikors, South Korea) for bone density and content analyses. The fracture strength at the midpoint of the femur and tibia was measured by using a universal testing machine (LR10K Plus, Lloyd Instruments Ltd., England) to record the maximum load weight based on the three-point bending method with an elongation rate of 10 mm/min.

### 2.6 Egg production and quality analysis

Dring the period of d 166–169 (approximately 24-week-old), all eggs of each cage (*n* = 8) were collected daily for analyzing egg production rate and broken egg ratio according to the formulas: Egg production rate (%) = (total eggs in each cage/the number of birds in each cage during the period) × 100%; and broken egg rate (%) = (total number of broken egg in each cage/total number of eggs in each cage) × 100%.

One egg from each of the 8 replicate cages (*n* = 8) within each group was randomly taken for analyzing egg quality. Egg weight, albumen height, yolk color, and Haugh unit were analyzed by an Egg Analyzer (EA-01, ORKA Food Technology Ltd., Israel). The eggshell color was determined using eggshell color reflectometer (QRS, TSS, England). After removing the contents and allowing the shells to dry, the thickness of eggshell was measured using a vernier caliper at three points around the equator of egg, and then the average value was calculated. Eggshell strength was tested by using an Egg Force Reader (EFR-01, ORKA Food Technology Ltd., Israel).

### 2.7 Statistical analysis

Regarding growth performance (sampling at d 110), egg production and bone performance (sampling at d 170) data, a 2-way ANOVA was used using the Generalized Linear Model (GLM) procedure of SPSS software (version 20.0, SPSS Inc., Chicago, IL, United States). The model included the main effects of BS, YCW, and their interaction. Tukey’s *post hoc* test was performed when a significant interaction was observed.

Considering that the 110- and 170-d birds originated from the same cage, a Repeated Measures ANOVA was used by the GLM procedure of SPSS software for data on immune status and intestinal morphology parameters (sampling at d 110 and 170). The model included the within-subjects variables (Time), between-subjects factors (BS and YCW) and their interaction. Tukey’s *post hoc* test was performed when a significant interaction was observed. Cage was the experimental unit for all variables data. Statistical significance was set at *p* < 0.05 for all data.

## 3 Results

### 3.1 Body weight and organ index

As shown in Table 1, birds fed with the diets containing YCW had a greater the thymus index than birds fed with the non-YCW diets at d 110 (*p* < 0.05). There were no interaction effects of BS and YCW supplements on birds’ body weight and other measured organ indexes (*p* > 0.05).

**Table 1:** Effects of *Bacillus Subtilis* (BS) and yeast cell wall (YCW) on body weight and organ index of 110-d hens.

Items		Body weight (kg)	Muscular stomach index (%)	Glandular stomach index (%)	Spleen index (%)	Thymus index (%)	Fasciola index (%)
Control		1.33	1.31	0.24	0.15	0.21	0.24
BS		1.36	1.20	0.23	0.15	0.18	0.23
YCW		1.38	1.16	0.23	0.13	0.23	0.18
BS + YCW		1.34	1.22	0.26	0.16	0.28	0.23
SEM		0.018	0.033	0.008	0.007	0.013	0.013
Main effects							
BS	−	1.36	1.22	0.24	0.14	0.22	0.21
	+	1.35	1.21	0.24	0.16	0.23	0.23
YCW	−	1.34	1.25	0.24	0.15	0.20^b^	0.23
	+	1.36	1.19	0.25	0.15	0.26^a^	0.20
*P*-value							
BS		0.941	0.681	0.778	0.283	0.702	0.462
YCW		0.519	0.331	0.576	0.704	0.018	0.249
BS × YCW		0.283	0.193	0.245	0.408	0.120	0.169

Values are represented by mean and SEM (*n* = 8 per group). ^a,b^ Means within a column with different letters differ at *p* < 0.05.

### 3.2 Serum inflammatory factors and immunoglobulin

Serum TNF-*α* concentrations were lower in 170-d laying hens compared with 110-d pullets (*p* < 0.05) (Table 2). Additionally, dietary supplementation with YCW increased the serum concentrations of IgM in hens (*p* < 0.05). Additionally, there was an interactive effect between YCW and age on serum IL-6 concentration of hens (*p* = 0.005). Specifically, serum IL-6 concentrations were lower in 170-d laying hens fed with YCW compared with 110-d pullets fed with YCW (*p* < 0.05) (Table 3).

**Table 2:** Effects of *Bacillus subtilis* (BS) and yeast cell wall (YCW) on serum inflammatory factors and immunoglobulin of 110- and 170-d hens.

Items		TNF-α (μg/L)	IL-6 (ng/L)	IgM (μg/L)
110 d	Control	7.27	2.46	8.78
	BS	7.39	2.90	8.75
	YCW	7.58	3.20	9.02
	BS + YCW	7.75	2.80	9.40
170 d	Control	7.23	2.85	8.42
	BS	7.24	2.79	8.70
	YCW	7.06	2.60	8.87
	BS + YCW	7.13	2.50	9.25
SEM		0.069	0.069	0.032
Within-subjects effects				
*P*-value	Time	0.017	0.099	0.073
	Time × YCW	0.082	0.005	0.708
	Time × BS	0.678	0.442	0.905
	Time × BS × YCW	0.345	0.155	0.945
Between-subjects effects				
*P*-value	YCW	0.477	0.553	0.006
	BS	0.536	0.952	0.109
	YCW × BS	0.791	0.118	0.488
Main effects				
BS	−	7.27	2.74	8.77
	+	7.36	2.78	9.03
YCW	−	7.28	2.75	8.66^b^
	+	7.35	2.77	9.14^a^
Time	110 d	7.49^a^	2.86	8.99
	170 d	7.16^b^	2.68	8.81

Values are represented by mean and SEM (*n* = 8 per group). ^a,b^ Means within a column with different letters differ at *P* < 0.05.

**Table 3:** Effect of time and YCW on serum IL-6 concentration in hens.

Time	YCW	IL-6 (ng/L)
110 d	−	2.68^ab^
	+	3.05^a^
170 d	−	2.82^ab^
	+	2.55^b^
SEM		0.060

Values are represented by mean and SEM (*n* = 16). ^a,b^ Means within a column with different letters differ at *p* < 0.05.

### 3.3 Intestinal morphology

BS supplementation increased jejunal and ileal villus heights, the ratio of V/C of the small intestine (duodenum, jejunum, and ileum), and decreased crypt depths in the duodenum and jejunum (*p* < 0.05) (Table 4; Supplementary Figures S1A,B). Lower duodenal crypt depth and jejunal villus height were observed in 170-d laying hens, whereas ileal villus height and the ratio of V/C increased with age (*p* < 0.05). An interactive effect was observed in the jejunal villus height between age and YCW (*p* = 0.022). Specifically, in the absence of YCW supplementation, jejunal villus height decreased with age (*p* < 0.05) (Table 5), whereas in the presence of YCW supplementation, jejunal villus height was not affected by increasing age (*p* > 0.05) (Table 5).

**Table 4:** Effects of *Bacillus subtilis* (BS) and yeast cell wall (YCW) on intestinal morphology of 110- and 170-d hens.

Items		Duodenum			Jejunum			Ileum		
		Villus height (μm)	Crypt depth, (μm)	V/C	Villus height (μm)	Crypt depth (μm)	V/C	Villus height (μm)	Crypt depth (μm)	V/C
110 d	Control	1586.06	119.32	13.42	1299.81	133.64	9.51	620.46	103.73	5.91
	BS	1610.55	125.09	13.96	1423.22	104.29	13.77	636.16	92.88	6.94
	YCW	1618.40	126.43	13.16	1226.20	117.46	10.64	606.78	86.18	7.21
	BS + YCW	1672.44	105.20	16.05	1256.32	110.02	11.46	651.33	86.66	7.58
170 d	Control	1447.75	127.50	11.64	1083.55	122.03	8.54	657.57	85.61	7.95
	BS	1566.11	95.53	16.49	1174.83	98.24	12.07	778.36	80.50	9.64
	YCW	1441.56	103.18	14.21	1160.64	110.38	11.09	691.14	86.50	8.11
	BS + YCW	1526.84	92.64	16.80	1291.11	102.93	12.98	798.95	86.09	9.30
SEM		30.979	3.517	0.505	25.040	3.376	0.384	18.273	1.916	0.241
Within-subjects effects										
*P*-value	Time	0.058	0.032	0.512	0.010	0.140	0.794	0.005	0.052	0.000
	Time × YCW	0.592	0.575	0.788	0.022	0.870	0.089	0.705	0.056	0.213
	Time × BS	0.631	0.297	0.305	0.709	0.794	0.899	0.229	0.754	0.380
	Time × BS × YCW	0.810	0.066	0.240	0.470	0.793	0.496	0.763	0.668	0.923
Between-subjects effects										
*P*-value	YCW	0.851	0.126	0.228	0.796	0.414	0.395	0.689	0.267	0.299
	BS	0.282	0.029	0.008	0.046	0.003	0.000	0.042	0.307	0.014
	YCW × BS	0.989	0.829	0.982	0.767	0.078	0.062	0.909	0.304	0.495
Main effects										
BS	−	1518.03	120.19^a^	12.96^b^	1208.79^b^	120.83^a^	10.00	646.81^b^	88.92	7.46^b^
	+	1593.98	104.37^b^	15.87^a^	1286.12^a^	103.92^b^	12.55	714.46^a^	86.52	8.34^a^
YCW	−	1538.40	120.09	13.39	1258.25	115.46	10.95	677.04	89.33	7.77
	+	1568.36	106.42	15.17	1230.32	110.90	11.42	685.82	86.37	8.03
Time	110 d	1625.81	118.69^a^	14.23	1297.98^a^	117.19	11.15	630.28^b^	90.75	7.05^b^
	170 d	1487.65	106.52^b^	14.53	1176.44^b^	108.77	11.22	729.77^a^	84.83	8.72^a^

Values are represented by mean and SEM (*n* = 8). ^a,b^ Means within a column with different letters differ at *P* < 0.05.

**Table 5:** Effect of time and YCW on the villus height of jejunum in hens.

Age	YCW	Villus height (μm)
110 d	−	1351.23^a^
	+	1239.89^ab^
170 d	−	1134.26^b^
	+	1218.63^ab^
SEM		25.040

Values are represented by mean and SEM (*n* = 16). ^a,b^ Means within a column with different letters differ at *p* < 0.05.

### 3.4 Production performance and egg quality

Dietary supplementation of BS significantly enhanced the eggshell thickness, albumen height, yolk color, and Haugh unit of hens (*p* < 0.05) (Table 6), while YCW increased the eggshell thickness and yolk color (*p* < 0.05). Additionally, there was a significant interaction between BS and YCW in egg weight (*p* < 0.05). Compared with the control bird, the heavier egg was observed in the BS, YCW, and BS + YCW birds (*p* < 0.05). However, no significant effect was observed in laying rate, broken egg rate, eggshell strength, and eggshell color of hens fed with BS and/or YCW (*p* > 0.05).

**Table 6:** Effects of *Bacillus subtilis* (BS) and yeast cell wall (YCW) on production performance and egg quality of 170-d hens.

Items		Laying rate (%)	Broken egg rate (%)	Egg weight (g)	Eggshell strength (kg)	Eggshell color	Eggshell thickness (mm)	Albumen height (mm)	Yolk color	Haugh unit
Control		80.83	7.08	52.08^b^	4.62	47.42	0.47	4.52	9.79	61.44
BS		85.42	3.33	55.26^a^	4.83	46.33	0.49	6.28	10.22	73.68
YCW		87.50	2.50	54.75^a^	4.66	45.60	0.49	5.83	10.25	70.06
BS + YCW		86.31	2.49	55.08^a^	4.94	45.45	0.50	6.88	10.32	77.81
SEM		1.961	0.862	0.328	0.081	0.362	0.022	0.342	0.063	2.440
Main effects										
BS	−	84.94	4.26	53.41	4.64	46.38	0.48^b^	5.17^b^	10.05^b^	65.75^b^
	+	85.90	2.88	55.17	4.89	45.89	0.49^a^	6.58^a^	10.27^a^	75.75^a^
YCW	−	83.33	5.04	53.67	4.73	46.80	0.48^b^	5.40	10.04^b^	67.56
	+	86.94	2.49	54.92	4.80	45.52	0.49^a^	6.35	10.28^a^	73.93
*P*-value										
BS		0.685	0.364	0.001	0.116	0.397	0.007	0.032	0.025	0.038
YCW		0.370	0.194	0.017	0.633	0.072	0.023	0.139	0.013	0.176
BS × YCW		0.492	0.367	0.007	0.818	0.522	0.413	0.576	0.101	0.629

Values are represented by mean and SEM (*n* = 8 per group). ^a,b^ Means within a column with different letters differ at *P* < 0.05.

### 3.5 Leg bone performance parameters

A significant interaction between BS and YCW was observed in the femoral length of 170-d hens (*p* = 0.003) (Table 7). The highest femur length among the treatments was observed in the BS birds (in the order, BS, 85.89 > YCW, 85.19 > BS + YCW, 83.44 > control, 82.64). Additionally, the supplementation of YCW increased the tibial diameter of 170-d hens (*p* < 0.05). However, dietary supplementation with BS and/or YCW did not affect femoral and tibial maximum load, bone mineral density, and bone mineral content (*p* > 0.05).

**Table 7:** Effects of *Bacillus subtilis* (BS) and yeast cell wall (YCW) on bone parameters of 170-d hens.

Items		Femur					Tibia				
		Length (mm)	Diameter (mm)	Maximum load (N)	Bone mineral density (g/cm^2^)	Bone mineral content (g)	Length (mm)	Diameter (mm)	Maximum load (N)	Bone mineral density (g/cm^2^)	Bone mineral content (g)
Control		82.64^b^	7.67	166.51	0.30	2.60	120.19	6.17	131.39	0.25	3.39
BS		85.89^a^	8.11	170.43	0.31	2.69	120.83	6.29	137.44	0.27	3.64
YCW		85.19^ab^	8.19	167.16	0.31	2.78	120.89	6.64	140.57	0.26	3.65
BS + YCW		83.44^ab^	7.72	163.56	0.29	2.53	118.66	6.39	136.14	0.26	3.43
SEM		0.431	0.103	4.052	0.010	0.080	0.520	0.070	2.680	0.005	0.072
Main effects											
BS	−	83.92	7.93	166.86	0.30	2.69	120.52	6.39	135.67	0.26	3.52
	+	84.58	7.89	166.99	0.30	2.62	119.67	6.34	136.79	0.27	3.53
YCW	−	84.16	7.86	168.60	0.30	2.64	120.49	6.23^b^	134.42	0.26	3.51
	+	84.31	7.96	165.36	0.30	2.68	119.70	6.51^a^	138.21	0.26	3.54
*P*-value											
BS		0.340	0.939	0.985	0.710	0.619	0.456	0.586	0.482	0.391	0.929
YCW		0.951	0.740	0.717	0.817	0.946	0.489	0.029	0.884	0.993	0.866
BS × YCW		0.003	0.051	0.662	0.424	0.315	0.184	0.145	0.351	0.318	0.114

Values are represented by mean and SEM (*n* = 8 per group). ^a,b^ Means within a column with different letters differ at *P* < 0.05.

## 4 Discussion

It has been evidenced that the production performance of laying hens declined with age, primarily due to age-related degradation of physical and physiological characteristics, such as deterioration of the intestinal mucosal morphology and bone mineral metabolism (18). It is necessary to strengthen the intestinal function and bone status of laying hens during the rearing and laying periods. Commercial chicks acquire bacteria from the incubation and rearing environment to initially establish the diversity and composition of the gut microbiota, which contributes to gut and bone development (19). Early life (at 1-day-old) administration of dietary BS or YCW may increase egg quality via improving the intestinal and bone development and immune status during the early sexual maturity stage in pullets. The current results support the hypothesis that early establishment of healthy intestinal and healthy skeletal characteristics is particularly important to maintain an optimal egg production performance.

The results showed that BS, YCW, and their combination had no significant effects on body weight as well as egg production and broken egg rate, of hens. Similarly, Liu et al. (20) reported that continuous supplementation of BS C-3102 (3.0 × 10^5^ and 6.0 × 10^5^ CFU/g) to 25-week-old Xuefeng black-bone hens for 56 days did not affect egg production or egg mass. In contrast, a low dose of BS DSM29784 (1.1 × 10^8^ CFU/kg) has been reported to improve Shaver White laying hens’ performance and egg quality from 19 weeks to 48 weeks of age (10). These inconsistent outcomes may be attributed to the different sources of BS and dose as well as the differences in breed and age of laying hens used in these studies. YCW is a green additive rich in *β*-glucan, mannan, and other bioactive substances with a variety of physiological functions (21). However, there are few studies exploring the direct effect of YCW on the growth performance of young hens. In this study, no significant effect of YCW on the growth performance of young hens was found. In contrast, a study reported that the inclusion of YCW extracts in mycotoxin-challenged chickens had beneficial effects for egg production and egg weight (22). Interestingly, the present experimental results show that the addition of YCW to the diets effectively modulates the immune function with significant changes in serum IL-6, and IgM as well as the thymus index. Parallel reports indicate that YCW supplementation can also enhance immune capabilities in hens when confronted with lipopolysaccharides challenges, consequently reducing inflammatory responses (23). It is speculated that these effects are attributed to the capacity of YCW on influencing the balance of gut microbiota, particularly on bacterial species associated with immune function (23). BS or YCW Modulating gut microbiota composition was not investigated in this study, but previous studies have reported that the supplements of both probiotic BS (24, 25) and YCW (23) at early life improve the host immunity responding to various inflammation and infection via modulating the profiles of gut microbiota and gene expression as well as pathomorphological characteristics.

The small intestine not only plays a crucial role in nutrient digestion and absorption but also serves as a vital barrier against pathogenic microorganisms. Elongated villus height increases the contact area of intestinal mucosa and chyme, by which it improves nutrient and mineral absorptive efficiency (26). Crypts are essential for the maintenance and functional renewal of villi, and crypt depth correlates with the rate of villus renewal (27). During pathophysiological processes such as natural epithelial shedding, inflammatory responses, or developing and repairing intestinal barrier integrity, villus renewal may be accelerated. Intestinal V/C ratio has become a common method used to evaluate intestinal function in nutrient absorption under various diet regimens and damaging challenges in animals including chickens (28, 29). A reduction in villus height coupled with an increase in crypt depth, leading to declined intestinal V/C ratios, consequently diminished nutrient absorptive efficiency, thereby negatively impacting overall performance (30). Conversely, an increase in villus height and or a decrease in crypt depth are typically associated with enhanced nutrient absorption efficiency (31). In current study, it was observed that the dietary supplementation of BS led to a notable decline in the intestinal crypt depth with increased V/C ratios in both the small intestine (the duodenum, jejunum, and ileum). This finding aligns with the study by Chen et al. (32), who reported that dietary addition of BS increased jejunal villus height and the V/C ratios, resulting in improved egg production and egg yield. In one of our previous studies, BS inhibited intestinal inflammation and oxidative stress through modulating intestinal flora and associated metabolites in 48-week-old laying hens, but without significant effect on intestinal histology (33). So early supplementation with BS, as a probiotic, in laying hens may be more beneficial to the intestinal development with long-lasting effect on egg production. On the other hand, YCW can function as a prebiotic to enhance intestinal barrier function by modulating the balance of immune responses and the gut microbiota (34, 35). Mechanistically, YCW upregulates the expression of signaling pathways related to intestinal stem cell proliferation and differentiation, such as Lgr5 and Wnt/*β*-catenin (35). Intestinal stem cells not only maintain the integrity of the intestinal epithelium through differentiation and proliferation, but also participate in the regulation of the immune and microbial environment of the intestine. Additionally, a well-developed intestinal structure increases the absorption of nutrients as well as minerals, including intestinal calcium ions, which are crucial for eggshell thickness and egg quality.

Egg quality and production performance are closely linked to intestinal absorption function. The current results showed that BS and YCW increased egg quality including egg weight, eggshell thickness, albumen height, yolk color, and Haugh unit, respectively. However, BS and YCW did not affect eggshell strength. Eggshell strength is not only dependent on the mineral content but also dependent on the contents of inorganic mineral ionic crystals (36). Additionally, feeding BS increased albumen height, a key indicator of egg albumen quality associated linked to egg quality (37). The greater albumen height observed in the group fed with BS supplementation may be attributed to enhanced protein absorption from the feed (38, 39). Overall, the current results reveal that BS and YCW supplementations significantly improve egg quality.

The better intestinal structure associated with the better function may favor bone performance in laying hens (40, 41). Generally, the demand for calcium in laying hens during growth with the gradually increasing of egg production. Approximately, 25–40% of the eggshell calcium is from the skeletal store (42). To meet the calcium requirements for eggshell formation, calcium reserved in the medullary bone is rapidly turned over at a rate of 1.6 g/day after the onset of sexual maturity (43). However, previous studies have shown that reliance on bone calcium can disrupt the balance between bone formation and resorption, potentially leading to osteoporosis characterized by reduced bone thickness and strength (43, 44). Therefore, ensuring young hens to achieve ideal peak bone mass and strength is a useful strategy to prevent osteoporosis in later age. However, no significant improvement in bone mineral density and content was observed in this experiment, which may be attributed to the fact that laying hens in this study were in the early stage of egg production and thus had a lower demand for bone calcium. There was a significant interaction effect of BS and YCW on the femoral length, and YCW significantly increased the tibial diameter at early stage laying hens. The medullary bones of the tibia and femur serve as a major source of calcium used in egg production (45). The longer or larger tibia and femur of hens may have larger calcium reserved to meet metabolic calcium demands, especially when reaching peak egg production. Yan et al. (46) reported that BS supplementation could promote bone growth in broilers under heat stress by inhibiting inflammation. These results may indicate that the beneficial effects of BS as well as YCW on bone development may more pronounced under various special conditions, such as heat stress (46) and age-related osteoporosis (47). The hypothesis will be validated in the further studies.

In conclusion, feeding BS and/or YCW during pullet to lay period could significantly improve both the egg and eggshell quality in early stage laying hens by boosting the intestinal development and modulating immunity with some skeletal benefits. BS and YCW combination did not show sufficient synergistic effects at the current conditions. The results provide novel insights into further investigating the effects of BS, YCW, and their interaction on production, health, and welfare of laying hens under various life cycles and stressful conditions.

## Data Availability

The original contributions presented in the study are included in the article/Supplementary material, further inquiries can be directed to the corresponding authors.

## References

[1] Broom LJ. The sub-inhibitory theory for antibiotic growth promoters. Poult Sci. (2017) 96:3104–8. doi: 10.3382/ps/pex114, PMID: 28595312

[2] Salim HM Huque KS Kamaruddin KM Beg M. Global restriction of using antibiotic growth promoters and alternative strategies in poultry production. Sci Prog. (2018) 101:52–75. doi: 10.3184/003685018X15173975498947, PMID: 29467062 PMC10365203

[3] Chandra RP Azraf A Anwar HM Kabir JI Christopher RR Lal SS. Prebiotics, probiotics and postbiotics for sustainable poultry production. World Poultry Sci J. (2021) 77:825–82. doi: 10.1080/00439339.2021.1960234, PMID: 40101104

[4] Kim YB Park J Lee HG Song JY Kim DH Ji W. Dietary probiotic Lacticaseibacillus paracasei NSMJ56 modulates gut immunity and microbiota in laying hens. Poult Sci. (2024) 103:103505. doi: 10.1016/j.psj.2024.103505, PMID: 38359769 PMC10877954

[5] Raval SD Archana G. Evaluation of synbiotic combinations of commercial probiotic strains with different prebiotics in in vitro and ex vivo human gut microcosm model. Arch Microbiol. (2024) 206:315. doi: 10.1007/s00203-024-04030-3, PMID: 38904672

[6] Hamprakorn K Maneewan B Jantasin W Lani MN Moonmanee T Panatuk J. Effect of extracted phycocyanin by-products as a synbiotic supplement on the production performance and intestinal morphology of broilers. Vet World. (2025) 18:52–9. doi: 10.14202/vetworld.2025.52-59, PMID: 40041502 PMC11873382

[7] Duc le H Hong HA Barbosa TM Henriques AO Cutting SM. Characterization of Bacillus probiotics available for human use. Appl Environ Microbiol. (2004) 70:2161–71. doi: 10.1128/AEM.70.4.2161-2171.2004 15066809 PMC383048

[8] Diaz-Sanchez S D'Souza D Biswas D Hanning I. Botanical alternatives to antibiotics for use in organic poultry production. Poult Sci. (2015) 94:1419–30. doi: 10.3382/ps/pev014, PMID: 25743421

[9] Tsai MY Shih BL Liaw RB Chen WT Lee TY Hung HW. Effect of dietary supplementation of *Bacillus subtilis* TLRI 211-1 on laying performance, egg quality and blood characteristics of Leghorn layers. Anim Biosci. (2023) 36:609–18. doi: 10.5713/ab.22.0274, PMID: 36634665 PMC9996276

[10] Neijat M Shirley RB Barton J Thiery P Welsher A Kiarie E. Effect of dietary supplementation of *Bacillus subtilis* DSM29784 on hen performance, egg quality indices, and apparent retention of dietary components in laying hens from 19 to 48 weeks of age. Poult Sci. (2019) 98:5622–35. doi: 10.3382/ps/pez324, PMID: 31222316

[11] Chen P Lv H Du M Liu W Che C Zhao J. *Bacillus subtilis* HW2 enhances growth performance and alleviates gut injury via attenuation of endoplasmic reticulum stress and regulation of gut microbiota in broilers under necrotic enteritis challenge. Poult Sci. (2024) 103:103661. doi: 10.1016/j.psj.2024.103661, PMID: 38547540 PMC11000119

[12] Zhang G Wang H Zhang J Tang X Raheem A Wang M. Modulatory effects of *Bacillus subtilis* on the performance, morphology, cecal microbiota and gut barrier function of laying hens. Animals. (2021) 11:1523. doi: 10.3390/ani11061523, PMID: 34073794 PMC8225007

[13] Kyoung H Kim E Cho JH Lee H Kim Y Park KI. Dietary yeast cell wall enhanced intestinal health of broiler chickens by modulating intestinal integrity, immune responses, and microbiota. Poult Sci. (2023) 102:102660. doi: 10.1016/j.psj.2023.102660, PMID: 37043955 PMC10140172

[14] M'Sadeq SA Wu SB Choct M Forder R Swick RA. Use of yeast cell wall extract as a tool to reduce the impact of necrotic enteritis in broilers. Poult Sci. (2015) 94:898–905. doi: 10.3382/ps/pev035, PMID: 25762162

[15] Johnson CN Hashim MM Bailey CA Byrd JA Kogut MH Arsenault RJ. Feeding of yeast cell wall extracts during a necrotic enteritis challenge enhances cell growth, survival and immune signaling in the jejunum of broiler chickens. Poult Sci. (2020) 99:2955–66. doi: 10.1016/j.psj.2020.03.012, PMID: 32475430 PMC7597693

[16] Lu J Wang Q Wang KH Ma M Wang XG Guo J. Effects of energy restriction during growing phase on the productive performance of Hyline Brown laying hens aged 6 to 72 wk. Poult Sci. (2023) 102:102942. doi: 10.1016/j.psj.2023.102942 37566966 PMC10432841

[17] Chen P Xu T Zhang C Tong X Shaukat A He Y. Effects of probiotics and gut microbiota on bone metabolism in chickens: a review. Meta. (2022) 12:1000. doi: 10.3390/metabo12101000, PMID: 36295902 PMC9612127

[18] Fu Y Zhou J Schroyen M Zhang H Wu S Qi G. Decreased eggshell strength caused by impairment of uterine calcium transport coincide with higher bone minerals and quality in aged laying hens. J Anim Sci Biotechnol. (2024) 15:37. doi: 10.1186/s40104-023-00986-2, PMID: 38439110 PMC10910863

[19] Lyu Z Yuan G Zhang Y Zhang F Liu Y Li Y. *Anaerostipes caccae* CML199 enhances bone development and counteracts aging-induced bone loss through the butyrate-driven gut-bone axis: the chicken model. Microbiome. (2024) 12:215. doi: 10.1186/s40168-024-01920-y, PMID: 39438898 PMC11495078

[20] Liu X Peng C Qu X Guo S Chen JF He C. Effects of *Bacillus subtilis* C-3102 on production, hatching performance, egg quality, serum antioxidant capacity and immune response of laying breeders. J Anim Physiol Anim Nutr. (2019) 103:182–90. doi: 10.1111/jpn.13022, PMID: 30484908

[21] Liu Y Wu Q Wu X Algharib SA Gong F Hu J. Structure, preparation, modification, and bioactivities of β-glucan and mannan from yeast cell wall: a review. Int J Biol Macromol. (2021) 173:445–56. doi: 10.1016/j.ijbiomac.2021.01.125, PMID: 33497691

[22] Weaver AC Weaver DM Adams N Yiannikouris A. Meta-analysis of the effects of yeast cell wall extract supplementation during mycotoxin challenges on the performance of laying hens. Toxins. (2024) 16:17. doi: 10.3390/toxins16040171, PMID: 38668596 PMC11054775

[23] Zhou J Fu Y Qi G Dai J Zhang H Wang J. Yeast cell-wall polysaccharides improve immunity and attenuate inflammatory response via modulating gut microbiota in LPS-challenged laying hens. Int J Biol Macromol. (2023) 224:407–21. doi: 10.1016/j.ijbiomac.2022.10.133, PMID: 36270403

[24] Lin D Song Q Zhang Y Liu J Chen F Du S. *Bacillus subtilis* attenuates hepatic and intestinal injuries and modulates gut microbiota and gene expression profiles in mice infected with schistosoma japonicum. Front Cell Dev Biol. (2021) 9:766205. doi: 10.3389/fcell.2021.766205, PMID: 34869360 PMC8635066

[25] Li G Tong Y Xiao Y Huang S Zhao T Xia X. Probiotic Bacillus subtilis contributes to the modulation of gut microbiota and blood metabolic profile of hosts. Comp Biochem Physiol C Toxicol Pharmacol. (2023) 272:109712. doi: 10.1016/j.cbpc.2023.109712, PMID: 37544638

[26] Gao H Zhao X Guo Y Li Z Zhou Z. Coated sodium butyrate and vitamin D(3) supplementation improve gut health through influencing intestinal immunity, barrier, and microflora in early-stage broilers. J Sci Food Agric. (2024) 104:4058–69. doi: 10.1002/jsfa.13288 38270478

[27] Bonis V Rossell C Gehart H. The intestinal epithelium - fluid fate and rigid structure from crypt bottom to villus tip. Front Cell Dev Biol. (2021) 9:661931. doi: 10.3389/fcell.2021.661931 34095127 PMC8172987

[28] Wilson FD Cummings TS Barbosa TM Williams CJ Gerard PD Peebles ED. Comparison of two methods for determination of intestinal villus to crypt ratios and documentation of early age-associated ratio changes in broiler chickens. Poult Sci. (2018) 97:1757–61. doi: 10.3382/ps/pex349, PMID: 29351670

[29] La J Raghunathan K Silvester JA Thiagarajah JR. ViCE: an automated and quantitative program to assess intestinal tissue morphology. J Pathol Inform. (2024) 15:100397. doi: 10.1016/j.jpi.2024.100397, PMID: 39435455 PMC11492601

[30] Belote BL Soares I Sanches AWD de Souza C Scott-Delaunay R Lahaye L. Applying different morphometric intestinal mucosa methods and the correlation with broilers performance under Eimeria challenge. Poult Sci. (2023) 102:102849. doi: 10.1016/j.psj.2023.102849, PMID: 37454643 PMC10384655

[31] Yang J Zhan K Zhang M. Effects of the use of a combination of two Bacillus species on performance, egg quality, small intestinal mucosal morphology, and cecal microbiota profile in aging laying hens. Probiotics Antimicrob Proteins. (2020) 12:204–13. doi: 10.1007/s12602-019-09532-x, PMID: 30810908

[32] Chen JF Xu MM Kang KL Tang SG He CQ Qu XY. The effects and combinational effects of Bacillus subtilis and montmorillonite on the intestinal health status in laying hens. Poult Sci. (2020) 99:1311–9. doi: 10.1016/j.psj.2019.11.016, PMID: 32111307 PMC7587652

[33] Zou XY Zhang M Tu WJ Zhang Q Jin ML Fang RD. Bacillus subtilis inhibits intestinal inflammation and oxidative stress by regulating gut flora and related metabolites in laying hens. Animal. (2022) 16:100474. doi: 10.1016/j.animal.2022.100474, PMID: 35220172

[34] Lee J Goo D Sharma MK Ko H Shi H Paneru D. Effects of graded yeast cell wall supplementation on growth performance, immunity and intestinal development of broiler chickens raised in floor pens for 42 days. Poult Sci. (2025) 104:104695. doi: 10.1016/j.psj.2024.104695, PMID: 39721260 PMC11732452

[35] Guo F Qiao J Hu Z Huang J Bi R Abbas W. Yeast cell wall polysaccharides accelerate yet in-feed antibiotic delays intestinal development and maturation via modulating gut microbiome in chickens. J Anim Sci Biotechnol. (2025) 16:14. doi: 10.1186/s40104-024-01145-x, PMID: 39856758 PMC11763161

[36] Wang S Zhang P Kong X Xie S Li Q Li Z. Delicate changes of bioapatite mineral in pig femur with addition of dietary xylooligosaccharide: evidences from Raman spectroscopy and ICP. Anim Sci J. (2017) 88:1820–6. doi: 10.1111/asj.12837, PMID: 28557169

[37] Chang X Wang B Zhang H Qiu K Wu S. The change of albumen quality during the laying cycle and its potential physiological and molecular basis of laying hens. Poult Sci. (2024) 103:104004. doi: 10.1016/j.psj.2024.104004, PMID: 39067125 PMC11331942

[38] Li Z Wang W Lv Z Liu D Guo Y. Bacillus subtilis and yeast cell wall improve the intestinal health of broilers challenged by *Clostridium perfringens*. Br Poult Sci. (2017) 58:635–43. doi: 10.1080/00071668.2017.1370697, PMID: 28841050

[39] DeHaan ER Thompson J Rusche WC de Jesus M Block E Rehberger T. Evaluation of long-term supplementation of a *Bacillus subtilis* direct-fed microbial and enzymatically hydrolyzed yeast cell culture product used alone or in combination on Clostridia, *Clostridium perfringens*, Escherichia coli, and Salmonella prevalence in beef steers. J Anim Sci. (2024) 102:skae156. doi: 10.1093/jas/skae156 38828876 PMC11196994

[40] Glisic M Boskovic M Baltic MZ Sefer D Markovic R. Performance, intestinal histomorphology and bone composition of broiler chickens fed diets supplemented with genistein. S Afr J Anim Sci. (2020) 50:241–52. doi: 10.4314/sajas.v50i2.7

[41] Sharma MK Regmi P Applegate T Chai L Kim WK. Osteoimmunology: a link between gastrointestinal diseases and skeletal health in chickens. Animals. (2023) 13:1816. doi: 10.3390/ani13111816, PMID: 37889704 PMC10251908

[42] Mueller WJ Schraer R Schraer H. Calcium metabolism and skeletal dynamics of laying pullets. J Nutr. (1964) 84:20–6. doi: 10.1093/jn/84.1.20, PMID: 14210016

[43] Whitehead CC Fleming RH. Osteoporosis in cage layers. Poult Sci. (2000) 79:1033–41. doi: 10.1093/ps/79.7.1033, PMID: 10901207

[44] Jin J Li Q Zhou Q Li X Lan F Wen C. Calcium deposition in chicken eggshells: role of host genetics and gut microbiota. Poult Sci. (2024) 103:104073. doi: 10.1016/j.psj.2024.104073, PMID: 39068697 PMC11339253

[45] Johnsson M Jonsson KB Andersson L Jensen P Wright D. Genetic regulation of bone metabolism in the chicken: similarities and differences to mammalian systems. PLoS Genet. (2015) 11:e1005250. doi: 10.1371/journal.pgen.1005250, PMID: 26023928 PMC4449198

[46] Yan FF Wang WC Cheng HW. *Bacillus subtilis*-based probiotic promotes bone growth by inhibition of inflammation in broilers subjected to cyclic heating episodes. Poult Sci. (2020) 99:5252–60. doi: 10.1016/j.psj.2020.08.051, PMID: 33142440 PMC7647906

[47] Yang HJ Zhang T Yue Y Jeong SJ Ryu MS Wu X. Protective effect of long-term fermented soybeans with abundant *Bacillus subtilis* on glucose and bone metabolism and memory function in ovariectomized rats: modulation of the gut microbiota. Food Secur. (2023) 12:2958. doi: 10.3390/foods12152958, PMID: 37569228 PMC10418888

