# Supplementary material for: Effect of supplementation continuously in pullet and early lay period with Bacillus subtilis and yeast cell wall on the intestinal morphology, bone parameters, and egg quality of hens
Source: Front Vet Sci. 2025 Apr 24;12:1584627. doi: 10.3389/fvets.2025.1584627 (PMC12058862; doi:10.3389/fvets.2025.1584627)
Supplement: Supplementary file 1 [file Image_1.pdf]

Supplementary Material

1.1 Supplementary Figures

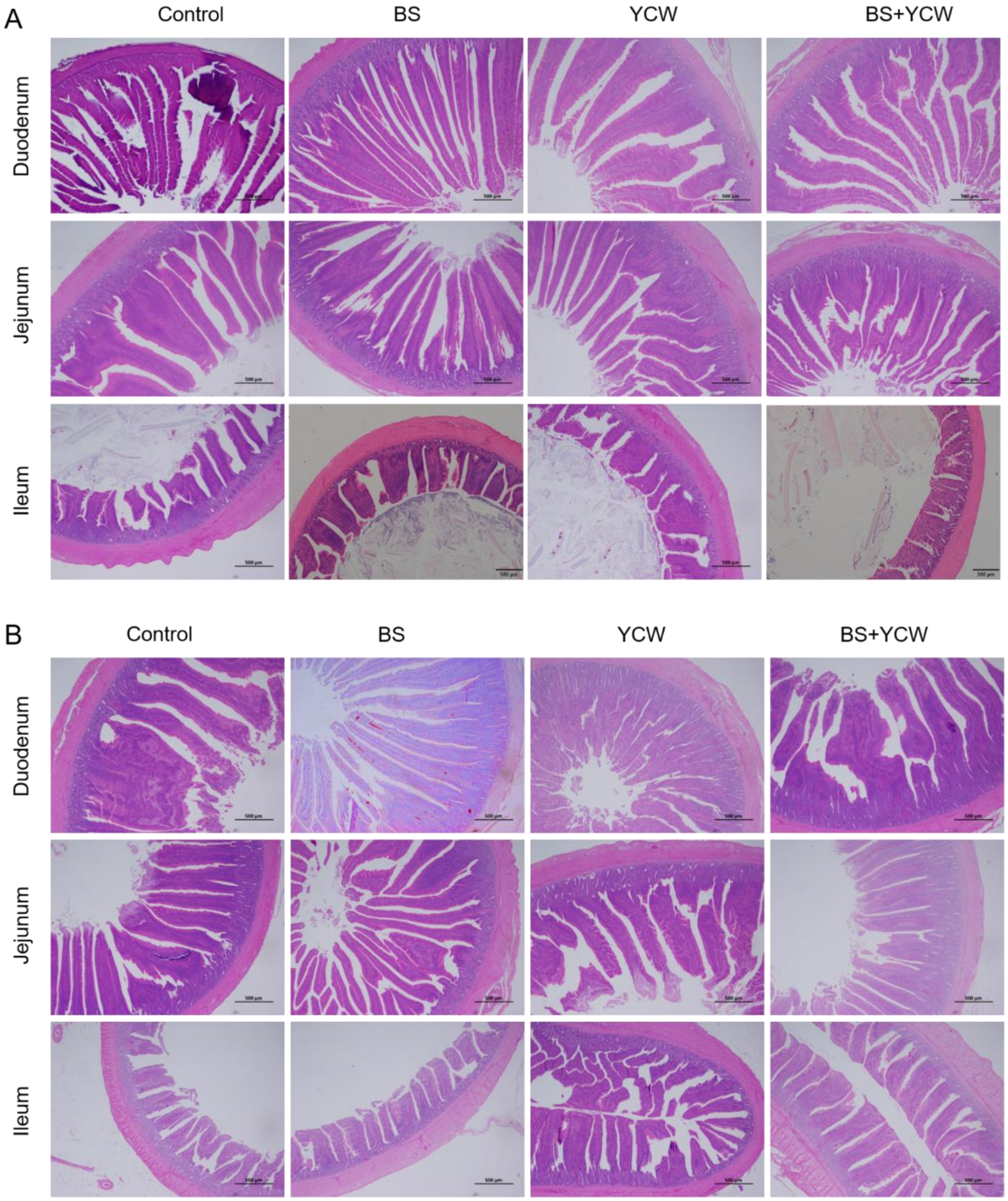

Supplementary Figure S1. Effects of *Bacillus subtilis* (BS) and yeast cell wall (YCW) on intestinal morphology of 110- and 170-d hens. (A) Representative images of hematoxylin and eosin-stained intestinal sections of 110-d pullets. (B) Representative images of hematoxylin and eosin-stained intestinal sections of 170-d hens. Scale bar = 500 µm. Abbreviations: Control, birds received regular

diet; BS, birds received regular diet and 0.5 g/kg of BS; YCW, birds received regular diet and 2 g/kg of YCW; BS+YCW, birds received regular diet and 0.5 g/kg of BS and 2 g/kg of YCW.
